# Facile Scale-Up of the Flow Synthesis of Silver Nanostructures Based on Norrish Type I Photoinitiators

**DOI:** 10.3390/molecules28114445

**Published:** 2023-05-30

**Authors:** Mahzad Yaghmaei, Connor R. Bourgonje, Juan C. Scaiano

**Affiliations:** Department of Chemistry and Biomolecular Sciences, University of Ottawa, Ottawa, ON K1N 6N5, Canada; myagh014@uottawa.ca (M.Y.); cbour019@uottawa.ca (C.R.B.)

**Keywords:** photochemistry, Norrish Type I reaction, nanomaterials, flow chemistry

## Abstract

Silver nanoparticles have become one of the most commercially and industrially relevant nanomaterials of the 21st century, owing to their potent antibacterial properties, as well as their useful catalytic and optical properties. Although many methods have been explored to produce AgNPs, we favor the photochemical approach using photoinitiators to produce AgNPs, owing to the high degree of control over reaction conditions, and the generation of so-called AgNP ‘seeds’ that can be used as-is, or as precursors for other silver nanostructures. In this work, we explore the scale-up of AgNP synthesis using flow chemistry and assess the usefulness of a range of industrial Norrish Type 1 photoinitiators in terms of flow compatibility and reaction time, as well as the resulting plasmonic absorption and morphologies. We establish that while all the photoinitiators used were able to generate AgNPs in a mixed aqueous/alcohol system, photoinitiators that generate ketyl radicals showed the greatest promise in terms of reaction times, while also showing greater flow compatibility compared to photoinitiators that generate 𝛼-aminoalkyl and α-hydroxybenzyl radicals. These findings help to establish a guideline for adapting photochemical AgNP syntheses to flow systems, helping to improve the scalability of the method in one of the largest industries in nanomaterial chemistry.

## 1. Introduction

Numerous free radical photoinitiators have been designed based on the Norrish Type I reaction involving C-C 𝛼-cleavage of carbonyl compounds [1,2,3,4]. In selected cases, one of the radicals generated is a strong electron donor; such systems are of great interest for the production of metal nanoparticles as they produce reducing agents with excellent spatial and temporal control [1,5,6,7]. Further, an additional benefit is kinetic control, as the light source irradiance can be readily adjusted by either controlling the power supplied (dimming), or simply changing the source-sample distance.

**Scheme 1:**
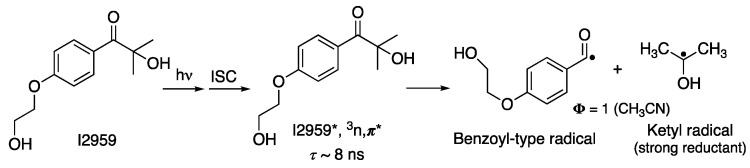
Mechanism for the decomposition of Irgacure 2959 (I-2859) showing the formation of the strongly reducing ketyl radical. Under typical reaction conditions the benzoyl radical yields the corresponding carboxylic acid that can behave as a mild nanoparticle stabilizer.

Many metal ions are excellent quenchers of excited states and frequently this quenching can limit the efficiency of photoinitiators. For these initiators to be effective, the Norrish Type I cleavage must be extremely fast and since most cleavages occur from the triplet state, this requires very short-lived triplet states [5]. A molecule that we have long recognized as a good nanoparticle precursor is Irgacure-2959 (I-2959), which has been widely employed in our group. I-2959 has a triplet lifetime of ~8 ns [3] and cleaves with a quantum yield of one in acetonitrile [8], Scheme 1.

Early work on the use of carbonyl compounds to generate metal nanoparticles frequently required very long exposure times [9], probably the result of the use of molecules with long-lived triplets that were readily quenched by the metal ions, typically Ag(I) or Au(III). Further, the need to generate strongly reducing radicals was not always recognized.

**Scheme 2:**
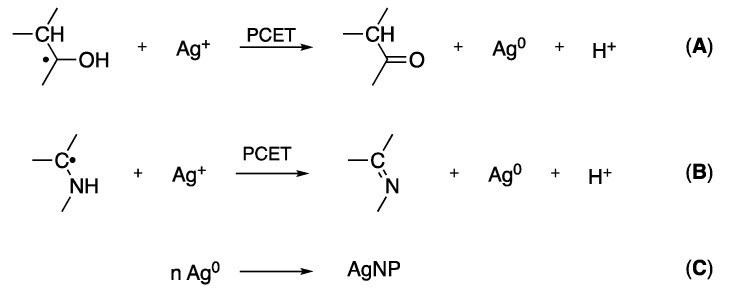
Electron transfer from ketyl and α-aminoalkyl radicals to reduce Ag(I) to Ag^0^, reactions A and B, respectively, which eventually generates silver nanoparticles (AgNP), reaction C.

Among reducing radicals, ketyl and α-aminoalkyl radicals are convenient sources, both operating by proton-coupled electron transfer reactions (PCET) and with commercial precursors readily available [1]. Scheme 2 illustrates the mechanism for silver; although, the same applies (with adjusted stoichiometry) for other ions, such as Au(III). Once the silver atoms have been produced, their next step is to form Ag2 [10], eventually leading to nanoparticle formation.

In this contribution, we examine several readily available radical photoinitiators frequently used in polymerization applications; although, our applications concentrate on the synthesis of nanostructures, illustrated here with silver seeds, small nanoparticles that we require for the production of other nanostructures with biological applications. We know that the morphology and size dispersity of nanostructures can depend on the precursor, including distinct byproducts, as well as on other additives, such as citrate that is of particular interest for our applications.

**Scheme 3:**
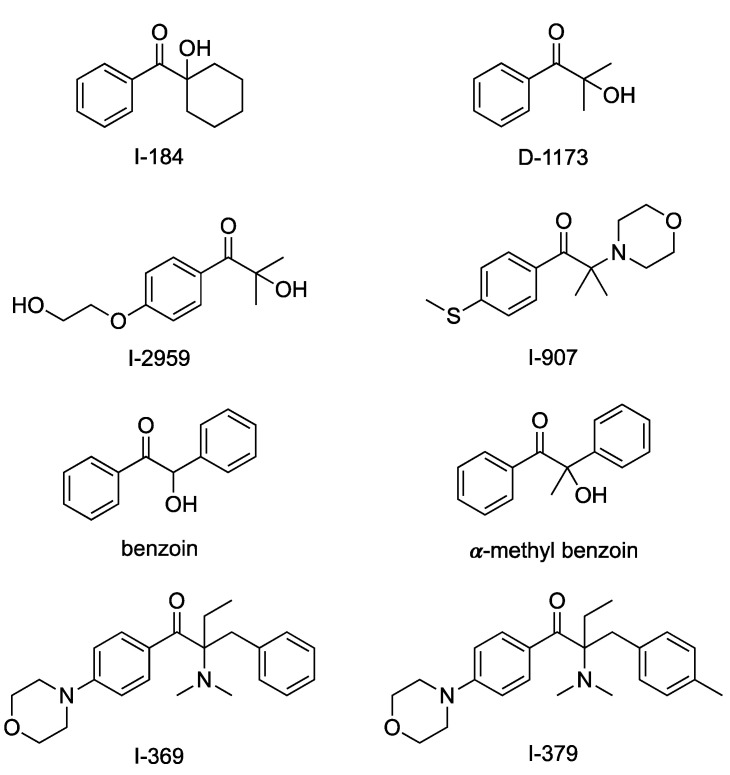
Structure of the molecules studies as precursors for AgNP. The letter ‘I’ is used as an abbreviation for Irgacure, and ‘D’ is used as an abbreviation for Darocur.

While nanoparticle synthesis is usually performed in batch reactions, in the last decade, there has been an interest in converting many reactions to flow systems, where scale-up is frequently easier and safety risks, if applicable, can be reduced [11,12]. In our laboratory, we have recently developed a flow actinometer that allows the quantification of the key reagent in photochemical reactions: light [8]. As we determined experimental irradiances in the UVA and UVB regions, we realized that our experimental geometry was exceptionally efficient in delivering photons to a flow reactor consisting of a narrow Teflon tube. In fact, in our preliminary work, we studied how short the residence time (<60 s) of the sample could be, and we discovered that our pumps were not fast enough to establish a threshold for quantitative Ag^+^ to Ag^0^ conversion. The answer to this issue is presented in the sections that follow. Scheme 3 shows the structures we tested.

## 2. Results and Discussion

This section is divided according to the methodologies used to synthesize nanoparticles and their characterization. The objective of this contribution is to establish a fast and reliable method to produce silver nanoparticles in a continuous flow. These small particles are sometimes described as ‘seeds’ and can be used to generate complex silver structures [13].

### 2.1. Spectroscopy of Initiators and Lamps Used

**Figure 1:**
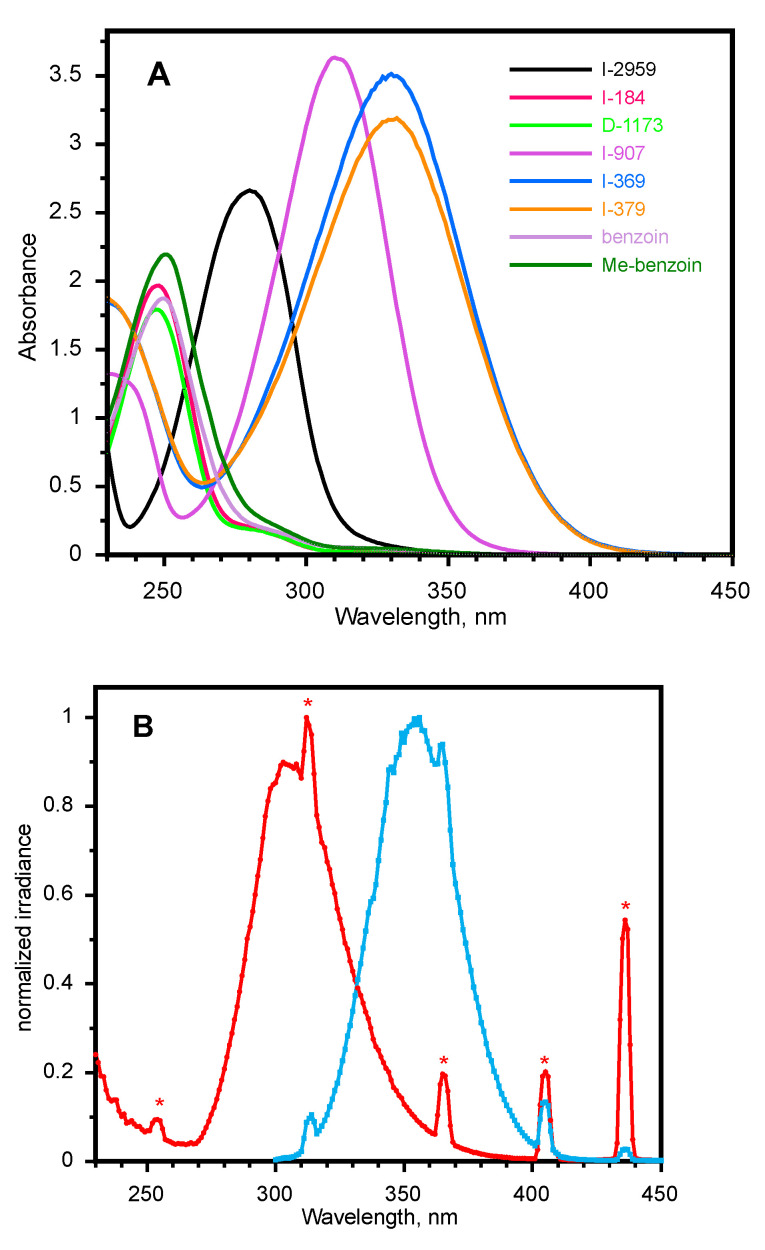
(**A**) Spectra of the initiators used at 0.2 mM in 1:1 aqueous methanol. All spectra in panel A were recorded using a Cary 7000 spectrometer that can handle high absorbances, as the Cary 60 instrument used elsewhere in this paper showed saturation at A > 2.7 in a 1 cm cuvette. The actual optical path under flow conditions is 0.15 cm and there is no saturation under these conditions. (**B**) Normalized spectra for the UVA (blue) and UVB (red) lamps utilized in this work. The sharp lines are leftovers (marked with ‘*’) from typical mercury emission lines at 254, 313, 365, 405, and 436 nm.

Our studies were performed with several photoinitiators that generate strongly reducing free radicals of the ketyl or 𝛼-aminoalkyl type with the structures shown in Scheme 3. They usually have strong absorptions in the UVA or UVB region and their spectra are shown in Figure 1A. The spectra for the UVA and UVB lamps used (mostly the latter) are shown in Figure 1B. The same wavelength scale is used for both to facilitate vertical comparisons.

### 2.2. Batch Experiments and Controls

**Figure 2:**
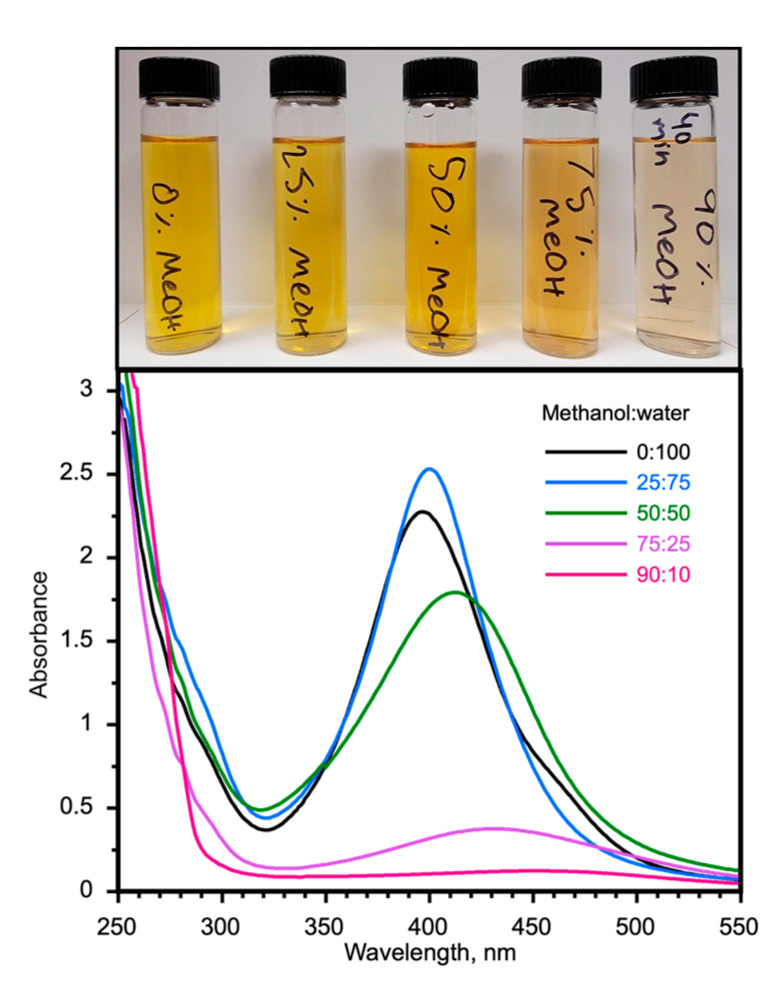
AgNPs synthesized in solutions containing different fractions of CH_3_OH ranging from 0% to 90%. Each reaction initially contains 1 mM citrate, 0.2 mM AgNO_3_, and 0.2 mM of I-2959. All samples were irradiated for 15 min in a photoreactor with 6 UVB lamps, except for the 90% CH_3_OH sample, which required 40 min to yield any noticeable color.

A number of preliminary batch experiments were performed in order to find out the preferred conditions for the flow work on which this contribution centers. In spite of the low concentrations required (frequently sub-millimolar) some of the initiators showed limited aqueous solubility, the exception being I-2959, where its glycol-like moiety facilitates solubility while also causing a red shift in the absorbance. Three co-solvents were tested: acetonitrile, ethanol, and methanol. The results with acetonitrile were disappointing as it seemed to slow down or inhibit nanoparticle formation, even at amounts as low as 10%, while the alcohols performed well. In the end, we settled on 50:50 methanol/water (*v*/*v*) as the preferred solvent (but not the only one) as it seemed compatible with all the initiators, with AgNO_3_ and with citrate used as a particle stabilizer. An example of solvent studies is presented in Figure 2 for I-2959 irradiated with UVB light for 15 min under an argon atmosphere; here, the Ag plasmon band seems best for 25% methanol; however, 50% still shows a well-resolved band and is a better choice when taking solubilities into consideration. Furthermore, when >50% MeOH was used, the absorption of the AgNP suspensions begin to drop considerably. For this reason, the reaction condition chosen for flow reactions going forward was 1:1 MeOH:H_2_O.

**Figure 3:**
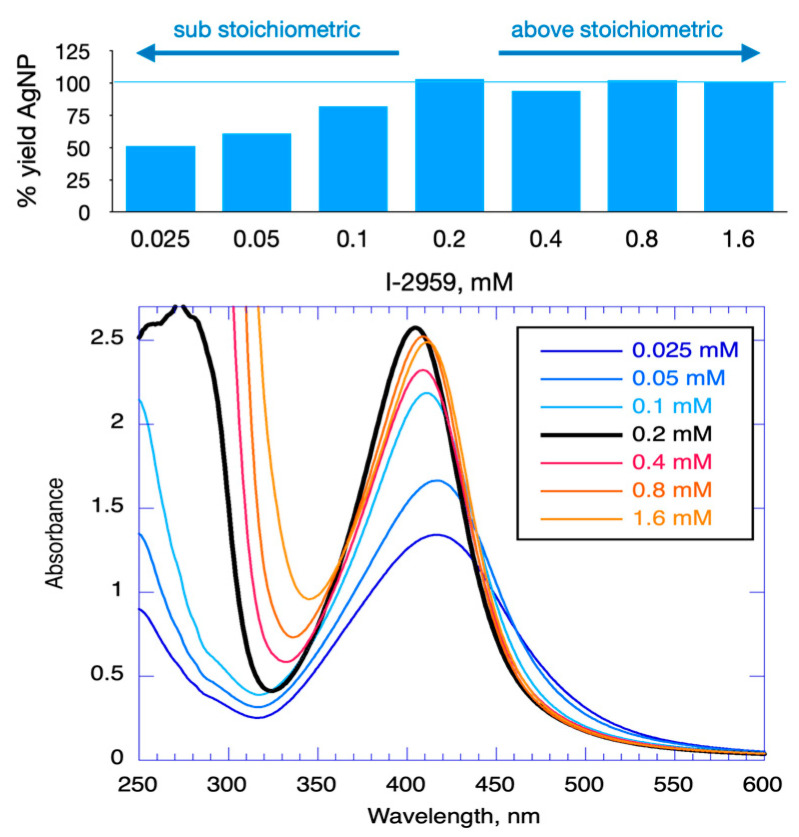
Effects of varying photoinitiator concentration in batch reaction. Top: Relative max absorption yield for AgNP suspensions in comparison to the stoichiometric ratio of I-2959 to AgNO_3_ for solutions with I-2959 concentrations ranging from 0.025 to 1.6 mM. Each reaction uses 0.2 mM of AgNO_3_, 1 mM of citrate, and 1:1 CH_3_OH:H_2_O. Solutions were irradiated for 15 min in a photoreactor with six 8W UVB bulbs. Bottom: Corresponding UV–Vis absorption spectra of AgNP plasmon bands with varying concentrations of I-2959. The black heavier line corresponds to the stoichiometric concentration of I-2959. Note that 0.1 mM initiator (light blue) generates 82% of the signal obtained with the stoichiometric concentration.

A very interesting observation from the batch experiments is that the initiator-to-Ag^+^ stoichiometric ratio required is less than one. If in Scheme 1 one assumes that only the ketyl radical leads to Ag^+^ reduction, then the minimum stoichiometric requirement would be 1.0. This is illustrated in Figure 3, where, for example, an 82% yield is obtained with 0.1 mM I-2959 and 0.2 mM Ag^+^, when the maximum anticipated yield would be 50% if only the ketyl radical is reactive. Clearly, the other materials in the reaction, citrate or methanol, may contribute to the reduction process. Citrate is known to be a modest reducing agent towards silver [14] and an established one in the case of gold [15]. In the case of methanol, the ĊH_2_OH radical is a good reducing agent, but its formation would require H-abstraction by the benzoyl radical of Scheme 1, a process that is expected to be slow, given the C-H bond dissociation energy of methanol [16], yet some contribution may be expected given that methanol is 12.4 M in the system.

Among the initiators in Figure 1, I-369 and I-379 are interesting because of their excellent absorbance match with the mission of the UVA lamp, something that may be advantageous in the presence of organic molecules (e.g., produced in catalytic processes) that are more likely to photodegrade with UVB than with UVA. However, this initiator presents consistency problems mentioned in the Supplementary Materials and addressed also in the flow experiments section.

Based on the results of bench experiments, it was decided to proceed using 0.2 mM of initiator, 0.2 mM of AgNO_3_, and 1 mM of trisodium citrate dissolved in 1:1 MeOH:H_2_O in order to best dissolve all of the initiators, while still producing AgNPs with strong, consistent plasmon bands.

Several other experiments were performed in batch and are described in the Supplementary Materials. In general, they show that the first three entries in Table 1 yield AgNP in a clean fast process with plasmon bands consistent with predominantly spherical particles, something confirmed in the TEM images (Figures S6–S13). Our best and most consistent results were obtained with initiators that yield ketyl radicals. The next section illustrates the excellent performance of ketyl radical generators under flow conditions.

### 2.3. Nanoparticle Synthesis Using Flow Strategies

**Figure 4:**
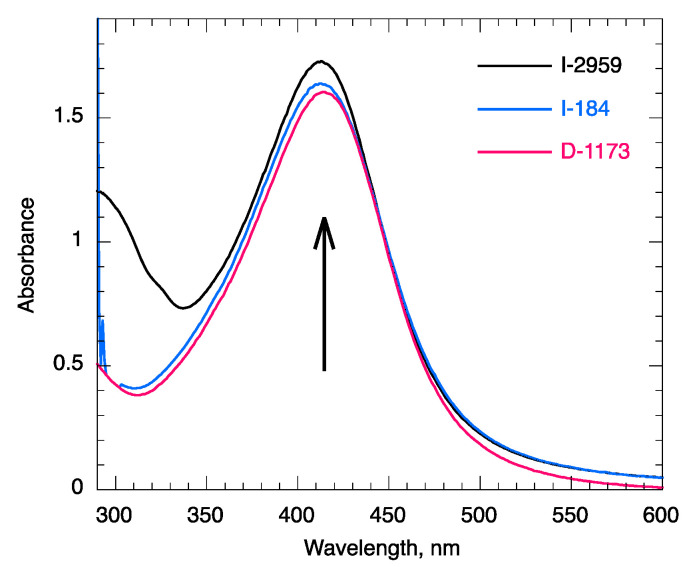
Comparison of initiators yielding ketyl radicals with high efficiency, produced under flow conditions with 0.2 mM of each photoinitiator, 0.2 mM of AgNO_3_, 1 mM of citrate, and 1:1 CH_3_OH:H_2_O and a residence time of 0.8 min. The arrow indicates growth during irradiation. The spike at 290 nm in the I-184 graph is due to bubbles in the flow.

The synthesis of AgNPs in flow was performed using the same experimental strategy as in a recent paper where we developed an actinometer for flow photoreactions [8]. Briefly, we utilize as light source 8 W fluorescent tubes from Luzchem, which are 30 cm long and with a diameter of 16 mm. The lamp is tightly wrapped with Teflon tubing with an outside diameter of 1.80 mm and an inside diameter of 1.50 mm corresponding to a wall thickness of 0.15 mm. The flow cavity in the tube is about 6.3 m long with a liquid capacity of 10 mL. The experimentally determined optical path is 0.15 cm [8]. The sample is supplied by a peristaltic pump with adjustable flow rate feeding from a container where the sample can be deaerated, normally with argon. At the exit, samples can be collected in vials or cuvettes, or be directly fed to a flow cell in a Cary-60 absorbance spectrometer. Particles undergo ripening over the next few hours in a process that can also depend on the presence or not of oxygen; however, the spectra shown below correspond to nascent particles under argon and the time lapse between the flow system and the spectrometer is usually less than one minute. We note at the onset that the most reliable initiators were those yielding ketyl radicals. This is illustrated in Figure 4, for a very short residence time, where all three initiators yield approximately the same plasmon band with comparable intensity.

**Figure 5:**
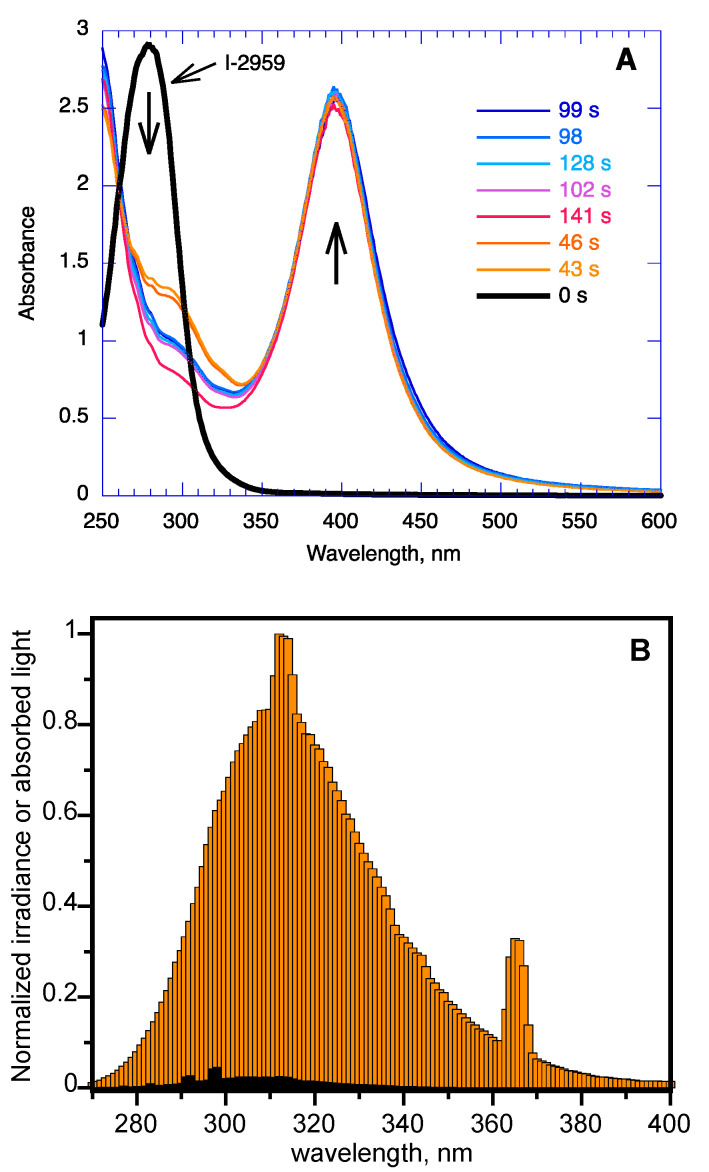
(**A**) Different residence times irradiated with UVB light and using I-2959 as initiator. Note that the residence times are not in increasing order, but rather are randomized; this is done to make sure any effects observed are not the result of a systematic change in the conditions. Silver nitrate (0.2 mM), sodium citrate (1 mM), I-2959 (0.2 mM) in water) purged with Argon and irradiated with a UVB lamp with different residence times (seconds) in 6.3 m Teflon tubing with a total irradiated volume of 10 mL. The arrow indicates growth during irradiation; (**B**) spectral distribution of the UVB lamp (normalized, orange) and the fraction of light absorbed by the I-2959 solution. (black), with the spectrum corrected for 0.15 cm optical path.

Figure 5 shows the spectra recorded for AgNP with different residence times; the remarkable observation is that a residence time of 40 s is enough to achieve the 100% conversion of a 0.2 mM Ag^+^ solution, this short residence time corresponds to the maximum flow speed for our pump and is equivalent to nearly one liter per hour. The reality is that we do not know how fast we could go and still have 100% conversion; however, it is interesting to note that the residence time of 0.8 min is much shorter than the required irradiation time of 15 min seen in the batch reaction, which yields only 6 mL of product.

The high yield contrasts with the fact that only 2.17% is being absorbed by I-2959 at a 0.2 mM concentration for the 1.5 mm optical path of the Teflon tubing. Intrigued by this result we used the actinometric determination method that we developed [8], to compare the chemical conversion of 0.2 mM with the light absorbed by the initiator (see our recent contribution on actinometry for detailed calculations [8]). In the shortest (40 s) residence time, the dose absorbed was 0.00012 Einstein L^−1^. This leads to a quantum yield for the Ag^+^ → Ag^0^ conversion of 1.67 ± 0.17. Clearly more than one Ag^+^ conversion per photon, but probably not high enough to suspect a chain reaction. We suspect that this is an autocatalytic process, just as it is in silver photography, where silver clusters lead to the reduction of neighboring Ag^+^ or salts in the presence of a reducing agent [17]. Possibly the UVB illumination contributes to this growth. The reducing agent can be methanol or citrate, more likely the latter. This is consistent with the results of batch experiments and illustrated in Figure 3. In general, I-2959, I-184, and D-1173 showed very similar behavior and the results obtained, along with those for other initiators are summarized in Table 1, with further details available in the Supplementary Materials. The photochemistry of some of these initiators has been examined in a classic publication from Turro’s group [3].

**Figure 6:**
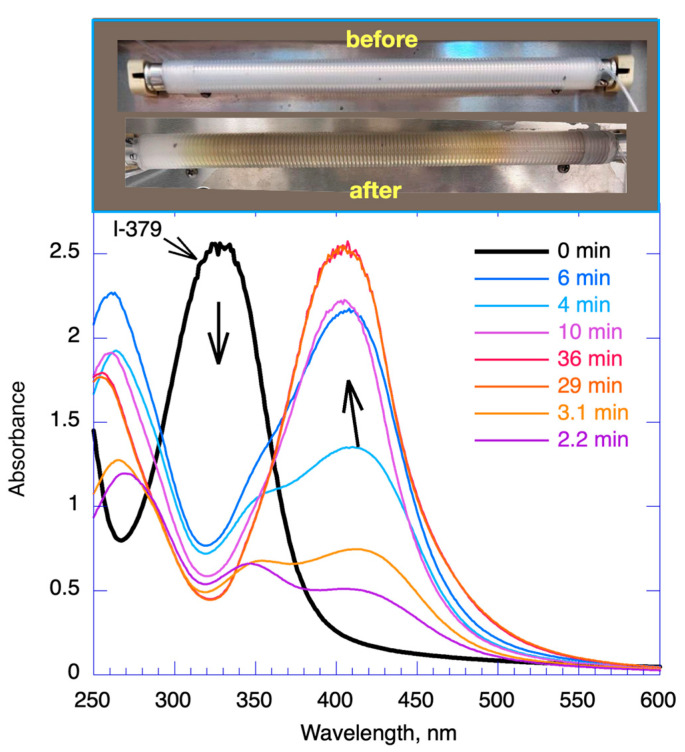
Different residence times irradiated with UVB light and using I-379 as initiator. Silver nitrate (0.2 mM), sodium citrate (1 mM), I-379 (0.2 mM) in 2:3 CH_3_OH:water purged Argon and irradiated with a UVB lamp with different residence times. Notice that the initiator (black with 𝜆_max_~323 nm is consumed in the first 2 min and has a slight blue shift at long irradiation times. The inset at the top shows the Teflon-wrapped lamp before and after experiment by using I-379 as an initiator. Notice the black deposits on the latter.

Another example with more complex photochemistry is shown in Figure 6 for I-379, where the initiator (𝜆_max_~325 nm) has a good overlap with the UVB lamp (see Figure 1) and is rapidly consumed, with essentially none left after 3.1 min. However, the AgNP plasmon band continues to grow and peaks at ~29 min. Once again, this suggests that the growth of the plasmon band reflects an autocatalytic process similar to that in silver photography [17], but stimulated by light. Notice also that the Teflon tubing (see top inset) develops black deposits after the flow tube has been used for 3 h. These deposits are believed to be silver or Ag_2_O formed during the prolonged exposure. We note that no deposits were observed when I-2959, I-184, or D-1173 were used as initiators. The deposits can be readily removed by flowing nitric acid and the Teflon tube can be reused.

The initiator I-369 showed behavior very similar to I-379, as expected, given the similarity of the two structures (see Table 1).

**Figure 7:**
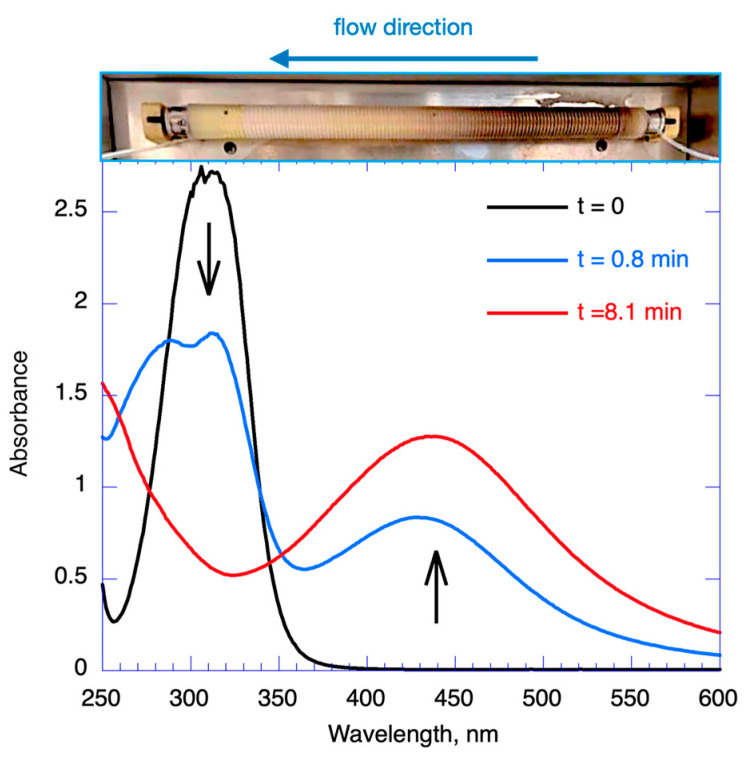
Different residence times irradiated with UVB light and using I-907 as initiator. Sample: Silver nitrate (0.2 mM), I-907 (0.2 mM), trisodium citrate (1 mM), and 1:1 CH_3_OH:water purged with argon. The inset at the top shows the Teflon-wrapped lamp after using I-907 as an initiator. Notice the black deposits, and in particular the dark areas are predominantly on the right side from which the reagents are fed to the system.

Initiator I-907 shows the best overlap with the UVB lamp (see Figure 1), producing strongly reducing α-aminoalkyl radicals, and the sulfur center should help stabilize nanoparticles. Its performance is good, but it produces nanostructures with a shifted plasmon band (~440 nm compared with the typical 395 nm), and the band is quite broad, Figure 7. The plasmon band stops growing after 8–10 min.

**Scheme 4:**
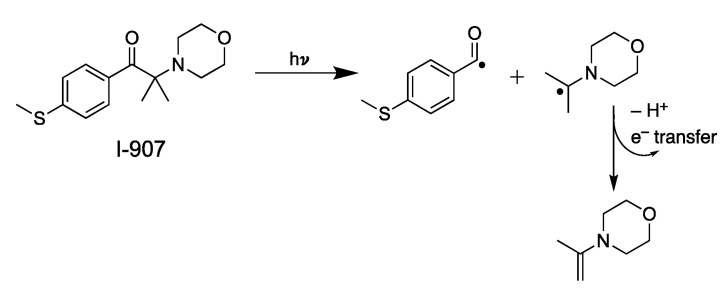
Anticipated Norrish Type I reaction of I-907 and chemistry of the α-aminoalkyl radical.

The photochemistry of I-907 generates reducing α-aminoalkyl radicals (Scheme 4). The benzoyl radical is expected to form the corresponding aldehyde or carboxylic acid, and both can stabilize AgNP, given the presence of a thioether.

Overall, I-907 is a reasonable initiator but produces particles with a red-shifted plasmon band and requires periodic nitric acid treatment of the Teflon tubing to clean up the silver deposits. This process is simple and fast, and the Supplementary Materials includes a Video S1 illustrating the cleaning process showing that the silver removal occurs as soon as the nitric acid front moves along the tube.

Finally, we come to the examples of benzoin and 𝛼-methylbenzoin. Benzoin is known to have a very short triplet state with a lifetime of around 1 ns [2,18]. Reports normally use non-aqueous solvents; although, measurements in methanol have been reported [2]. Benzoin has a rather low extinction coefficient, which becomes a disadvantage, for example, in polymer chemistry applications [4]. Frequently, the absorbance is enhanced by the addition of thioether substitution [3,4], something that is also evident when comparing the spectra of benzoin and I-907 in Figure 1. To the best of our knowledge, benzoin has not been studied in water.

**Figure 8:**
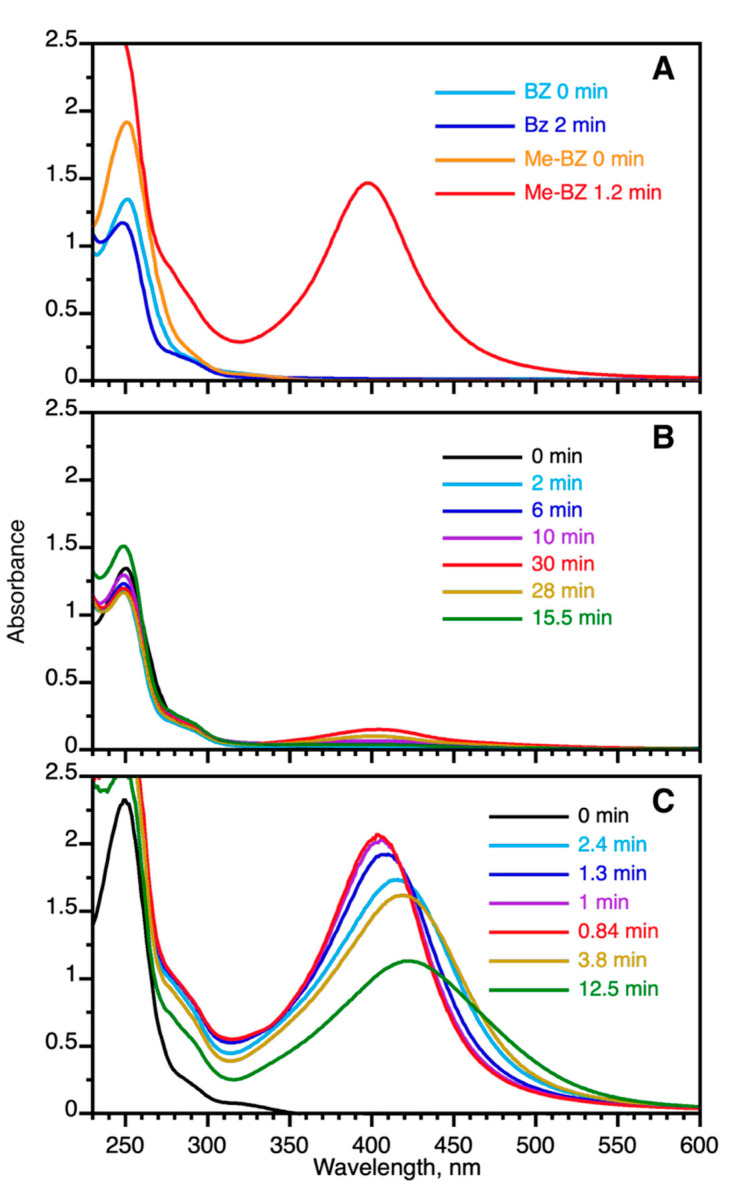
Benzoin as an initiator for spectra corresponding to different residence times in minutes. Silver nitrate (0.2 mM), benzoin (0.2 mM), and trisodium citrate (1 mM) were deaerated with argon and irradiated with UVB lamp in a flow system with 6.3 m Teflon tubing. The order shown in each panel for residence times corresponds to the sequence of acquisition, ensuring that no systematic order is used (**A**) in water comparing benzoin (totally inefficient) with 𝛼-methylbenzoin, the latter showing intermediate efficiency; (**B**) benzoin in water showing that at long residence times, there is a slow growth of the plasmon band; (**C**) spectra in 1:1 CH_3_OH:water. Notice that the top absorbance is nearly 30 times larger in panel (**C**), and further, the highest absorbance is achieved in ~1 min.

Unfortunately, benzoin proved to be a very inefficient initiator in water, but it was reasonably efficient in 50% aqueous methanol, see Figure 8. In contrast, 𝛼-methyl benzoin was very efficient, even in water (Figure 8A), while the absorption spectra of the two ketones are very similar, with 𝜆max~250 nm.

Even in 50% aqueous methanol, the performance is good. Notice that after 1.3 min, the main effect of irradiation is a red shift of the plasmon band and a gradual decrease in absorbance at the maximum. Overall, while benzoin seems a challenging initiator, 𝛼-methylbenzoin seems straightforward and quite efficient, even in water (Figure 1A). We speculated that benzoin in water may undergo some enolization, but the very limited aqueous solubility led to the failure of the NMR work aimed at detecting any enol formation. In the context of this work, which is aimed at practical recommendations for initiators for nanoparticle fabrication, benzoin is not recommended, while 𝛼-methylbenzoin is.

### 2.4. Concentration Tolerance and Stability during Long Fabrication Times

**Figure 9:**
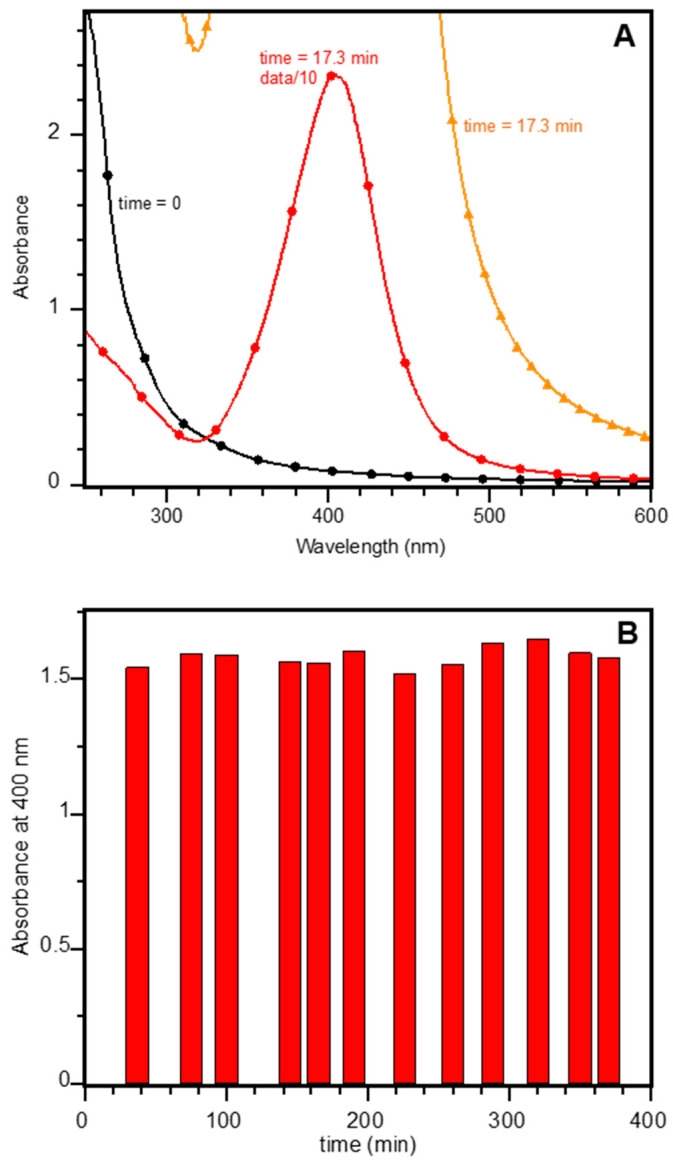
(**A**) For our concentration study, we used silver nitrate (2 mM), I-184 (2 mM), and trisodium citrate (10 mM), in MeOH 10%, in water deaerated with Ar, under UVB irradiation with a residence time of 17.3 min. The absorbance axis has been limited to the 2.7 specification for the Cary 60 spectrometer and the graph includes a sample diluted 10 times. (**B**) Monitored at 400 nm for over 6 h, with silver nitrate (0.2 mM) and all other parameters identical to panel (**A**).

We were also interested in establishing to what extent the continuous generation of particles in the flow system would offer consistent results. We were also interested in establishing if AgNP could be produced at higher concentrations, and then diluted just before use. For this work, we chose I-184, one of the highly reliable initiators. The results in Figure 9A confirm that a higher concentration of reagents can be readily utilized and dilution to the spectrometer absorbance range shows an excellent well-resolved plasmon band. In the context of continuous production of nanoparticle solutions Figure 9B, the 400 nm absorbance shows variations of ±2% over a 6 h period, illustrating the high reliability of the method.

### 2.5. Characterization of the Nanostructures Prepared

**Figure 10:**
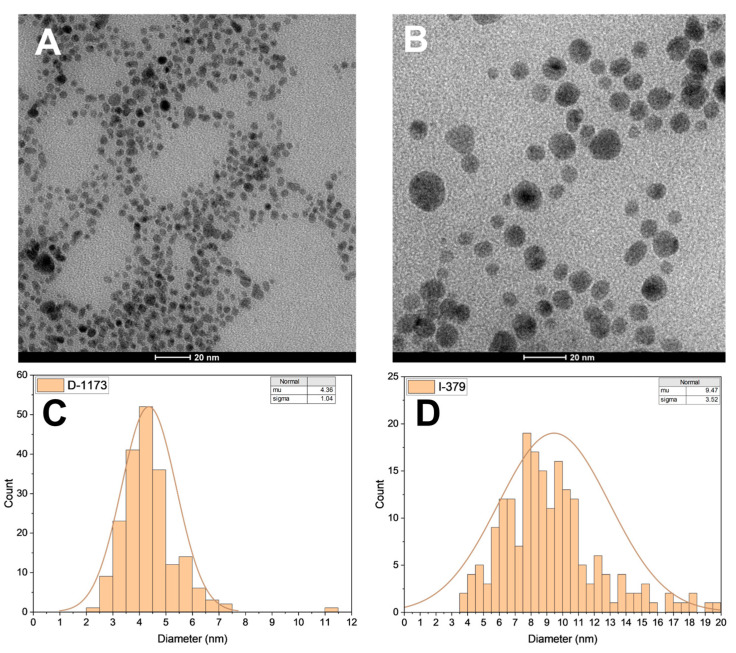
TEM image of AgNPs generated with D-1173 (**A**) and I-379 (**B**) synthesized in-flow using 50% MeOH and their corresponding particle size histograms ((**C**,**D**), respectively). Histograms were made from the counting of 200 particles. XRD for similarly stabilized particles is available in the literature [19].

AgNP morphologies for all the initiators were approximately spherical, with D-1173, I-2959, and I-907 producing seeds with the smallest size and narrowest size distributions (Table 2). Conversely, I-379 and I-369 yielded the largest seeds with broader size distributions. TEM images (Figure 10) and histograms for NPs made with each initiator are available in the Supplementary Materials, see Figures S6–S13, with corresponding UV–Vis spectra for each sample used for the TEM imaging shown in Figure S15.

## 3. Materials and Methods

### 3.1. Materials

Chemicals and materials were sourced from multiple suppliers for the experimental work. Ciba Specialty Chemicals supplied the initiators I-184, I-907, I-369, I-379, and D-1173, while Sigma Aldrich provided the benzoin. BASF supplied I-2959, and Alfa Aesar provided alpha-hydroxy-alpha-methylbenzyl phenyl ketone. Solvents were purchased from Fisher chemical.

### 3.2. Spectroscopy

The UVB lamp (8W) and Expo panel (EXPO-01) were obtained from Luzchem Research, Inc., Ottawa, Canada and Teflon tubing (STT-15) was procured from Component Supply Company, Sparta, TN, USA. The pump (minipuls 3) was purchased from Gilson.

UV–Vis spectroscopy was performed using a Cary 7000 UV–Vis spectrometer for bench reactions and for flow when needed; this instrument can measure high absorbances reliably. A Cary 60 UV–Vis spectrometer was normally used for flow reactions. Transmission electron microscopy (TEM) was performed using an FEI Tecnai G2 spirit Twin TEM microscope.

### 3.3. Batch Reactions

Batch reactions (without flow) were performed in 20 mL glass vials containing in a typical reaction 0.2 mM AgNO_3_, 0.2 mM photoinitiator, and 1 mM trisodium citrate dissolved in a 1:1 mixture of MeOH and water. Samples were deoxygenated under gentle argon bubbling for 20 min before being placed in a photoreactor containing 6 UVB lamps for 15 min. Samples were analyzed with UV–Vis spectroscopy and TEM as-prepared without any purification.

Similar preparations were also prepared using acetonitrile and ethanol as co-solvents. Notably, without the addition of co-solvent, most photoinitiators were not easily dissolved.

### 3.4. Flow Reactions

The light sources were 8 W fluorescent lamps from Luzchem installed on an EXPO panel from the same supplier. The Teflon tubing has an outside diameter of 1.80 mm and an inside diameter of 1.50 mm corresponding to a wall thickness of 0.15 mm. We have shown before that this setup leads to an experimental optical path of 0.15 cm [8].

## 4. Conclusions

The photochemical synthesis of silver nanoparticles under flow conditions has proven extremely efficient and reproducible, yielding suitable AgNP seeds under UVB irradiation with exposure times of less than one minute for the best initiators. The recommended initiators are those yielding ketyl radicals efficiently following Norrish Type I cleavage. Among the eight structures shown in Scheme 3, four of them are highly recommended, I-184, D-1173, α-methylbenzoin, and I-2959. The last of which showing the best absorption properties, adequate aqueous solubility, and is readily available. We anticipate that the seeds produced here will enable the synthesis of other morphologies with interesting biological applications [13,20].

## Figures and Tables

**Table 1:** Summary of flow photochemistry results of silver nanoparticle production with different initiators, using 0.2 mM of AgNO_3_, 0.2 mM of initiator, and 1 mM of citrate in deaerated solvent with UVB lamp (see Scheme 3).

Initiator	Solvent	𝜆max FWHM (nm)	Residence Time (min)	Notes
I-184	Water	400 50	0.8	Clean no deposits in the flow tube
	1:1 CH_3_OH:Water	420 100	4	
D-1173	Water	400 60	0.8	Clean no deposits in the flow tube
	1:1 CH_3_OH:Water	415 95	0.8	
I-2959	Water	400 50	0.7	Clean no deposits in the flow tube
	1:1 CH_3_OH:Water	415 80	0.8	
I-907	1:9 CH_3_OH:Water	445 160	4	Heavy black deposits in the flow tube
	1:1 CH_3_OH:Water	445 150	8	
Benzoin	Water	400 85	30	Very weak signals
	1:1 CH_3_OH:Water	410 80	0.8	Yellow deposits in the flow tube
α-Me Benzoin	Water	400 70	0.8	Yellow deposits in the flow tube
	1:1 CH_3_OH:Water	405 80	0.8	Faint yellow deposits in flow tube
I-369 ^a^	1:1 CH_3_OH:Water	410 90	4	Heavy black deposits in the flow tube
I-379	1:1 CH_3_OH:Water	410 90	6	Heavy black deposits in the flow tube
	2:3 CH_3_OH:Water	410 80	10	

^a^ I-369 results are the same for UVA and UVB light (Figure S14).

**Table 2:** Average diameters and standard deviation of AgNPs synthesized using each photoinitiator with optimized flow conditions. All values are based on the counting of 200 nanoparticles.

Initiator Used	Average Diameter (nm)	Standard Deviation (nm)
I-2959	4.6	1.8
D-1173	4.4	1.0
I-184	7.6	2.6
I-907	5.3	1.5
I-369	8.6	4.2
I-379	9.5	3.5
benzoin	6.1	2.2
α-methylbenzoin	8.3	2.7

## Data Availability

Original data are available from the corresponding author upon reasonable request.

## Supplementary Materials

The following supporting information can be downloaded at: https://www.mdpi.com/article/10.3390/molecules28114445/s1, Figures S1–S15: showing spectral data under different conditions, TEM images and the corresponding size histograms, and a schematic representation of the experimental setup. In addition, the Video “nitric acid cleanup” shows how the flow tube can be readily cleaned by flowing nitric acid.

## Sample Availability

Samples of the compounds are not available from the authors.

## References

[1] Scaiano J.C., Stamplecoskie K.G., Hallett-Tapley G.L. (2012). Photochemical Norrish type I reaction as a tool for metal nanoparticle synthesis: Importance of proton coupled electron transfer. Chem. Commun..

[2] Lipson M., Turro N.J. (1996). Picosecond investigation of the effect of solvent on the photochemistry of benzoin. J. Photochem. Photobiol. A Chem..

[3] Jockusch S., Landis M.S., Freiermuth B., Turro N.J. (2001). Photochemistry and Photophysics of a-Hydroxy Ketones. Macromolecules.

[4] Esen D.S., Arsu N., Da Silva J.P., Jockusch S., Turro N.J. (2013). Benzoin type photoinitiator for free radical polymerization. J. Polym. Sci. Part A Polym. Chem..

[5] McGilvray K.L., Decan M.R., Wang D., Scaiano J.C. (2006). Facile Photochemical Synthesis of Unprotected Aqueous Gold Nanoparticles. J. Am. Chem. Soc..

[6] Marin M.L., McGilvray K.L., Scaiano J.C. (2008). Photochemical Strategies for the Synthesis of Gold Nanoparticles from Au(III) and Au(I) Using Photoinduced Free Radical Generation. J. Am. Chem. Soc..

[7] Scaiano J.C., Billone P., Gonzalez C.M., Maretti L., Marin M.L., McGilvray K.L., Yuan N. (2009). Photochemical routes to silver and gold nanoparticles. Pure Appl. Chem..

[8] Yaghmaei M., Scaiano J.C. (2023). A simple Norrish Type II actinometer for flow photoreactions. Photochem. Photobiol. Sci..

[9] Miranda O.R., Ahmadi T.S. (2005). Effects of Intensity and Energy of CW UV Light on the Growth of Gold Nanorods. J. Phys. Chem. B.

[10] Stamplecoskie K.G., Scaiano J.C. (2011). Kinetics of the Formation of Silver Dimers: Early Stages in the Formation of Silver Nanoparticles. J. Am. Chem. Soc..

[11] Cambié D., Bottecchia C., Straathof N.J.W., Hessel V., Noël T. (2016). Applications of Continuous-Flow Photochemistry in Organic Synthesis, Material Science, and Water Treatment. Chem. Rev..

[12] Rehm T.H. (2020). Flow Photochemistry as a Tool in Organic Synthesis. Chem. Eur. J..

[13] Stamplecoskie K.G., Scaiano J.C. (2010). Light Emitting Diode Irradiation Can Control the Morphology and Optical Properties of Silver Nanoparticles. J. Am. Chem. Soc..

[14] Jiang X.C., Chen C.Y., Chen W.M., Yu A.B. (2010). Role of Citric Acid in the Formation of Silver Nanoplates through a Synergistic Reduction Approach. Langmuir.

[15] Turkevich J., Stevenson P.C., Hillier J. (1951). A Study in the Nucleation and Growth Processes in the Synthesis of Colloidal Gold. Discuss. Faraday Soc..

[16] Cruickshank F.R., Benson S.W. (1969). Carbon-hydrogen bond dissociation energy in methanol. J. Phys. Chem..

[17] Belloni J. (2002). The role of silver clusters in photography. Comptes Rendus Physique.

[18] Lewis F.D., Lauterback R.J., Heine H.G., Hartmann W., Rudolph H. (1975). Photochemical a-cleavage of Benzoin Derivatives. Polar Transition States for Free Radical Formation. J. Am. Chem. Soc..

[19] Mahy J.G., Kiendrebeogo M., Farcy A., Drogui P. (2023). Enhanced Decomposition of H_2_O_2_ Using Metallic Silver Nanoparticles under UV/Visible Light for the Removal of p-Nitrophenol from Water. Catalysts.

[20] Bourgonje C.R., Regis Correa da Silva D., McIlroy E., Calvert N.D., Shuhendler A.J., Scaiano J.C. (2023). Silver Nanoparticles with exceptional near-infrared absorbance for photo-enhanced antimicrobial applications. J. Mat. Chem B.

