# Supplementary material for: Facile Scale-Up of the Flow Synthesis of Silver Nanostructures Based on Norrish Type I Photoinitiators
Source: Molecules. 2023 May 30;28(11):4445. doi: 10.3390/molecules28114445 (PMC10254936; doi:10.3390/molecules28114445)
Supplement: Supplementary file 1 [file molecules-28-04445-s001.zip › molecules-2418807-supplementary/flow AgNP SI vf.pdf]

Supplemental Information

Facile scale-up of the flow synthesis of silver nanostructures based on Norrish Type I photoinitiators

Mahzad Yaghmaei [1†], Connor R. Bourgonje [1†] and Juan C. Scaiano[1,*]

[1] Department of Chemistry and Biomolecular Sciences, University of Ottawa, Ottawa, Ontario K1N 6N5, Canada

* jscaiano@uottawa.ca

† equal contribution.

Contents

Figure S1 2

Figure S2 3

Figure S3 4

Figure S4 5

Figure S5 6

Figure S6 7

Figure S7 8

Figure S8 9

Figure S9 10

Figure S10 11

Figure S11 12

Figure S12 13

Figure S13 14

Figure S14 15

Figure S15 16

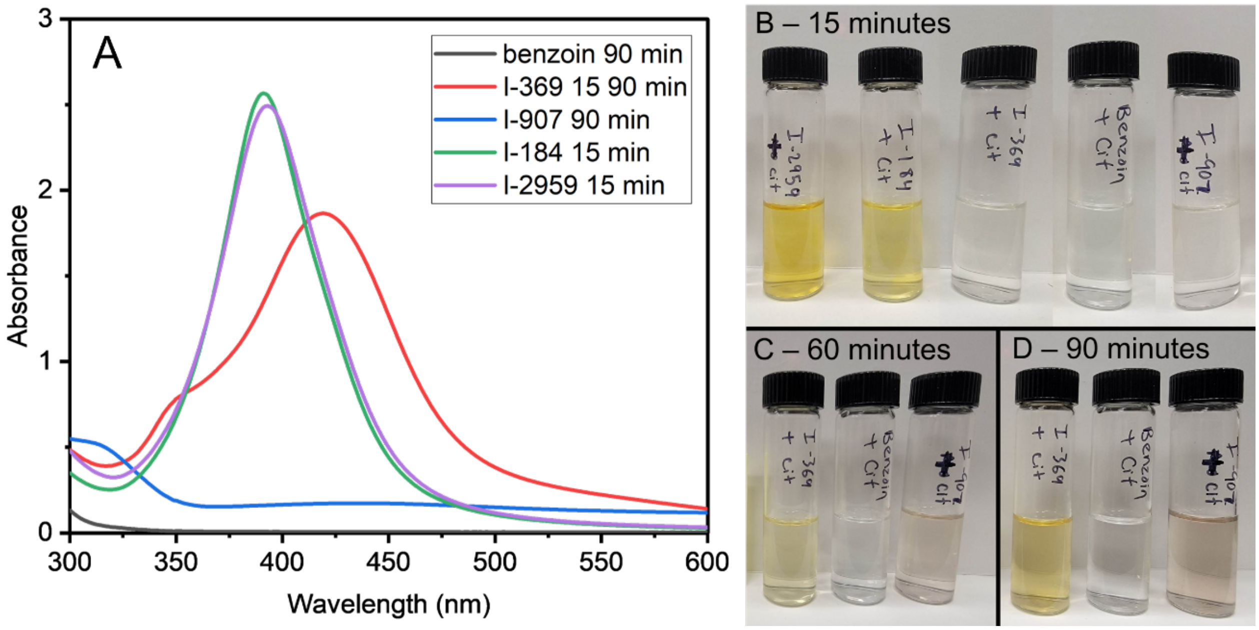

Figure S1**:** A) UV-Vis spectra of AgNP solutions produced using 0.2 mM I-2959, I-184, I-369, benzoin, or I-907 as photoinitiators in water. I-2959 and I-184 completed the reaction in 15 minutes, while I-369 required 90 minutes to generate similar levels of AgNP absorbance. Benzoin and I-907 did not yield any notable plasmonic absorbance peaks within 90 minutes of reaction. Corresponding appearance of the nanoparticle suspensions at 15 minutes (B), 60 minutes (C), and 90 minutes (D) are shown in the right panel. It should be noted that I-2959 dissolved easily

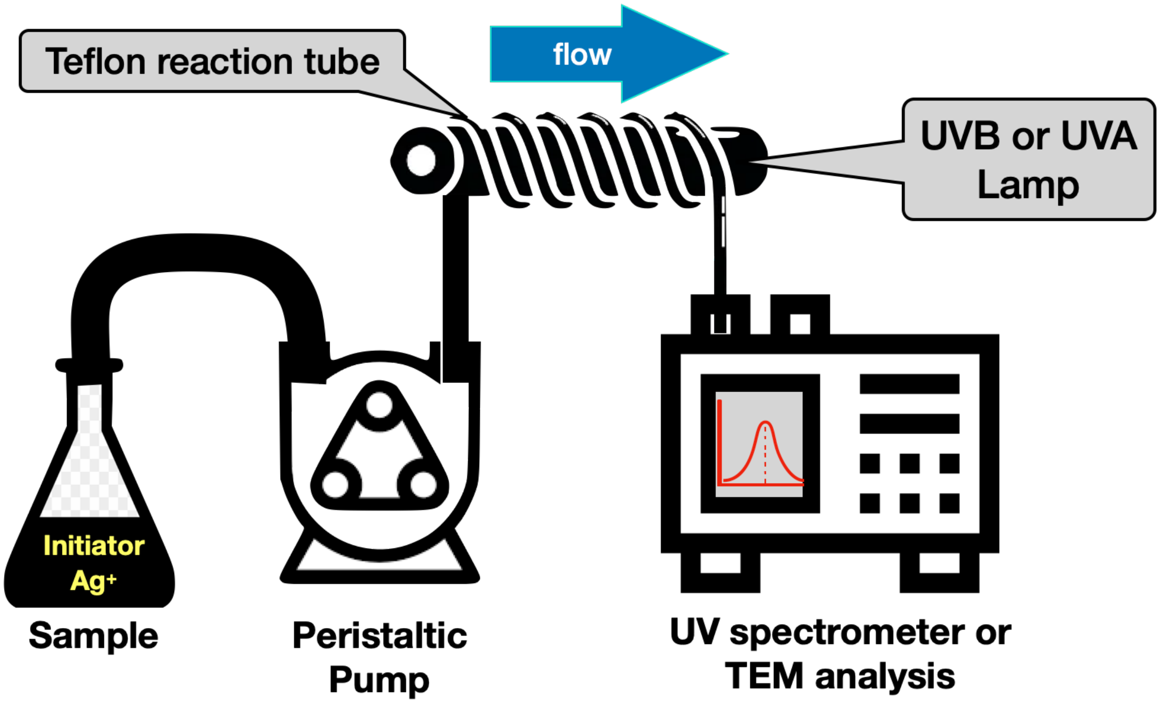

Figure S2: Schematic representation of the experimental flow system.

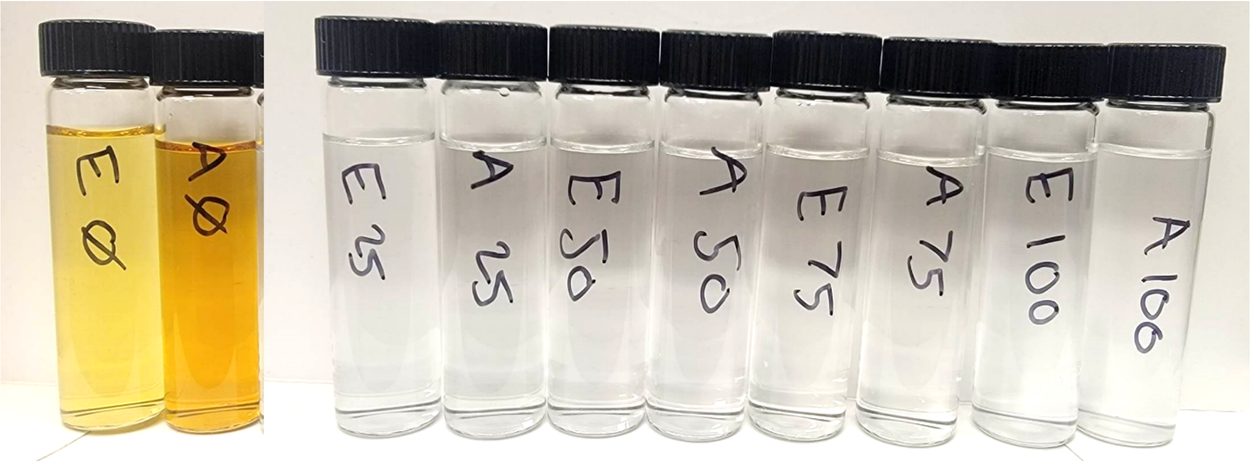

Figure S3**:** Appearance of solutions containing 0.2 mM AgNO3, 1 mM citrate, and 0.2 mM of either I-2959 (“A”) and I-184 (“E”) as initiators, in mixtures of MeCN and water (0%, 25%, 50%, 75% and 100% MeCN) following 15 minutes of irradiation with in a photoreactor with 6 8W UVA lamps (for the 0% MeCN samples). The samples containing MeCN showed no change after 15 minutes. The MeCN containing samples were subsequently irradiated for a total of 90 min, but still did not yield any AgNPs.

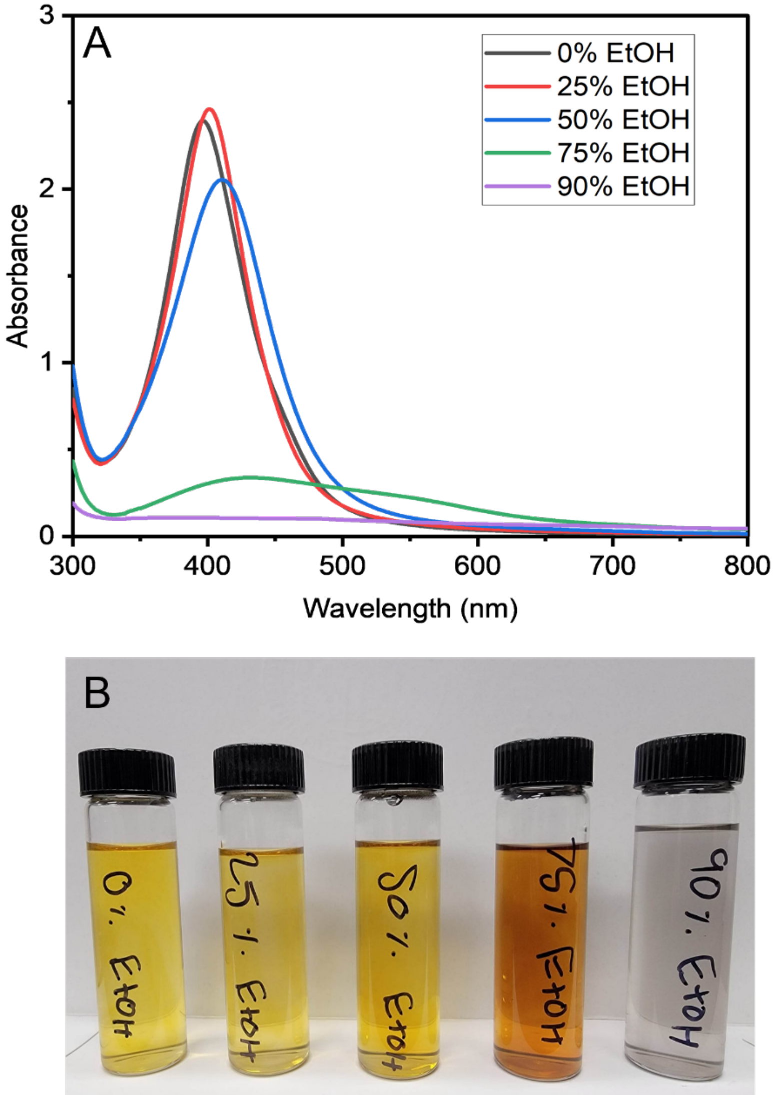

Figure S4: A) UV-Vis spectra of AgNPs synthesized in batch scale, using 0.2 mM I-2959 as an initiator. Solutions were prepared in mixtures of EtOH/H_2O in ratios ranging from 0 to 90% EtOH, and irradiated for 15 minutes in a photoreactor with 6 8W UVB lamps. The 90% EtOH sample was irradiated for an additional 25 mins, as little color could be seen after 15 minutes of irradiation. B) Corresponding appearance of AgNPs produced in solutions containing different fractions of EtOH following reacting under UVB.

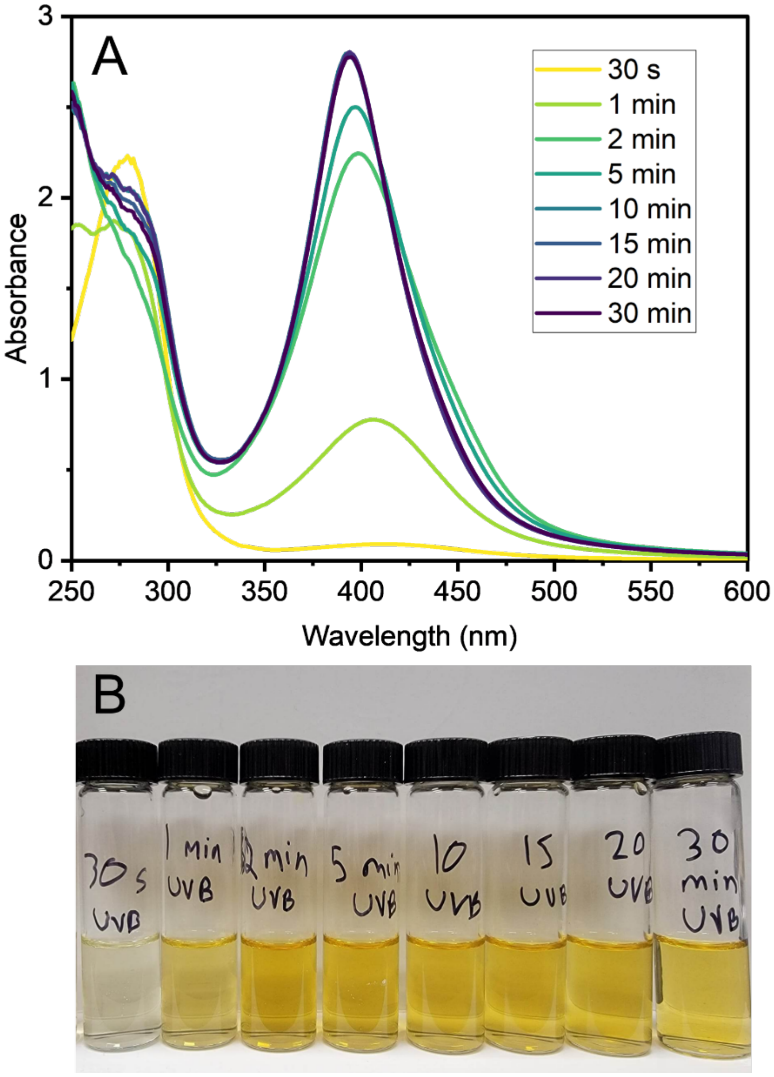

Figure S5: A) UV-Vis spectra of AgNPs synthesized in single batches, using 0.2 mM I-2959 in H_2O. Samples were irradiated in a photoreactor with 6 UVB lamps for 30 sec to 30 min to determine the endpoint of the reaction. B) The appearance of the corresponding nanoparticle suspensions.

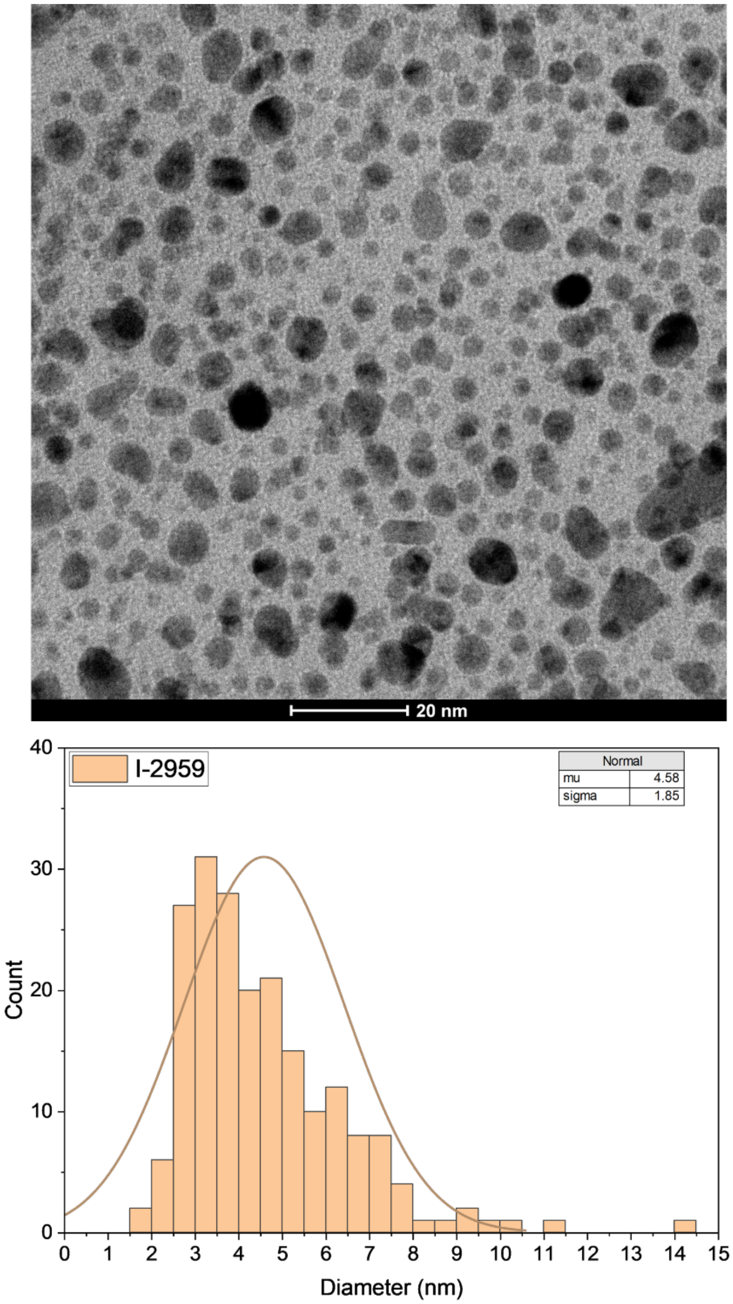

Figure S6**:** Top: Representative TEM image of AgNPs generated using the optimized flow reaction conditions using I-2959 as a photoinitiator. Bottom: Corresponding histogram for I-2959 generated AgNPs, n = 200 particles. Inset lists average size, mu, and standard deviation, sigma, in nm.

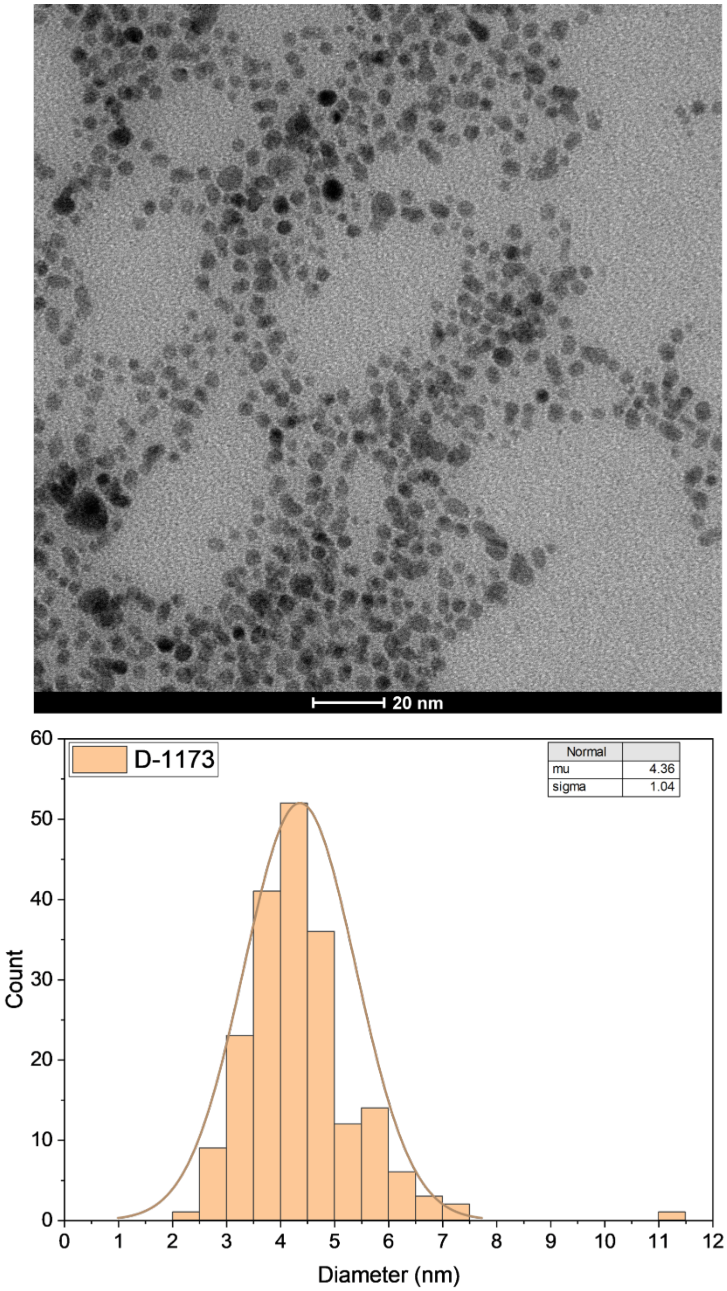

Figure S7**:** Top: Representative TEM image of AgNPs generated using the optimized flow reaction conditions using D-1173 as a photoinitiator. Bottom: Corresponding histogram for D-1173 generated AgNPs, n = 200 particles. Inset lists average size, mu, and standard deviation, sigma, in nm.

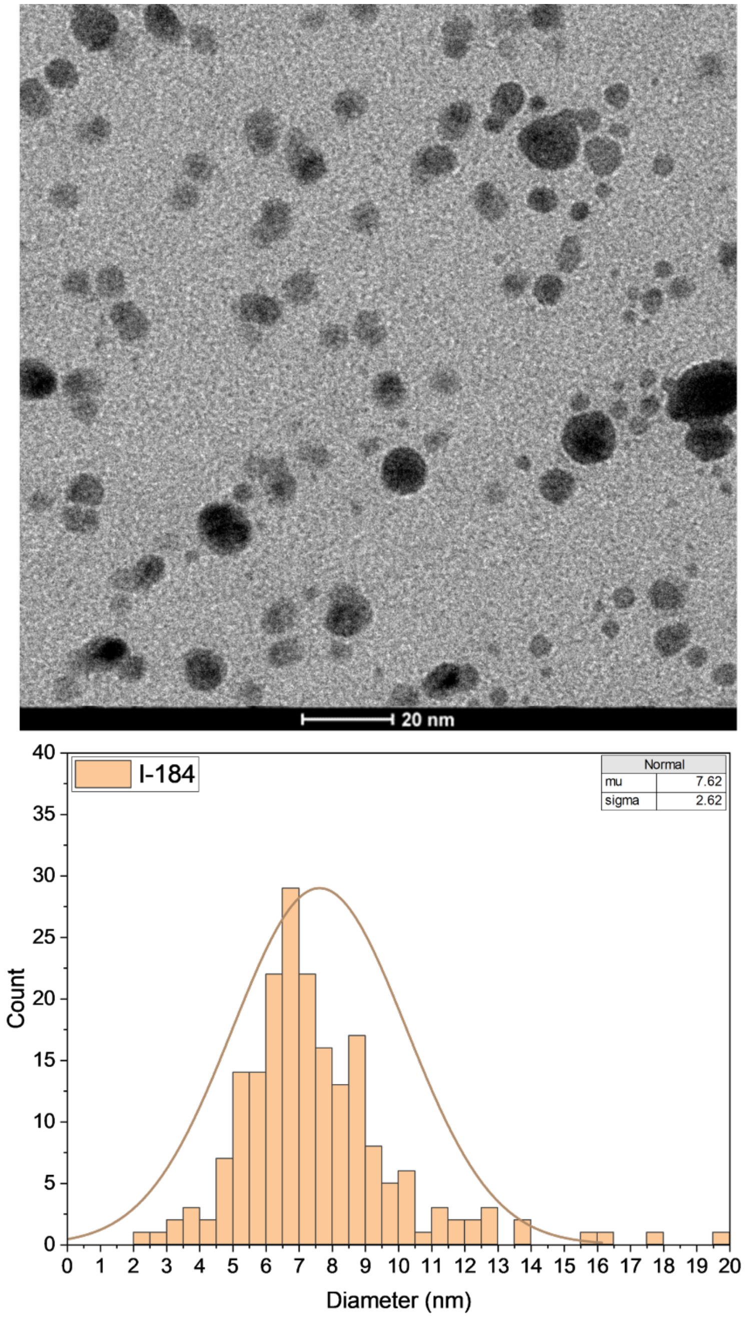

Figure S8**:** Top: Representative TEM image of AgNPs generated using the optimized flow reaction conditions using I-184 as a photoinitiator. Bottom: Corresponding histogram for I-184 generated AgNPs, n = 200 particles. Inset lists average size, mu, and standard deviation, sigma, in nm.

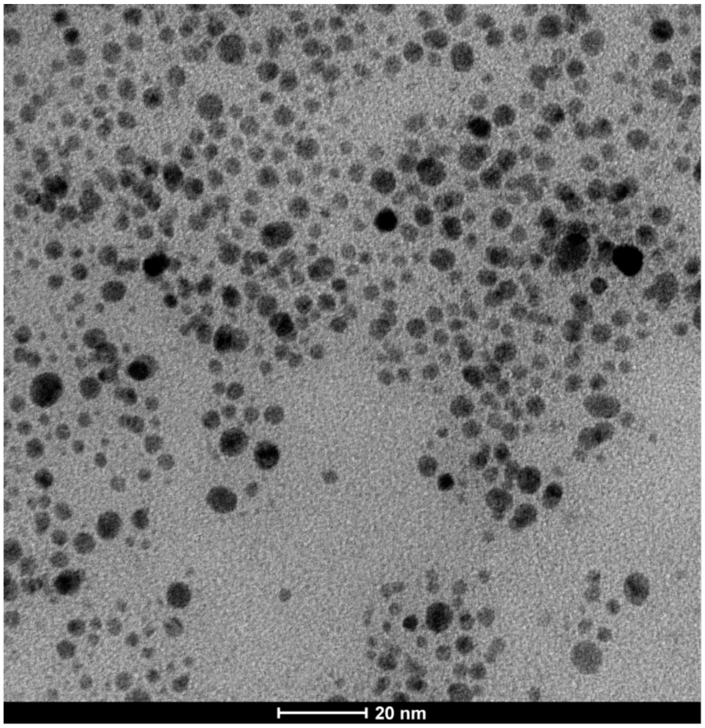

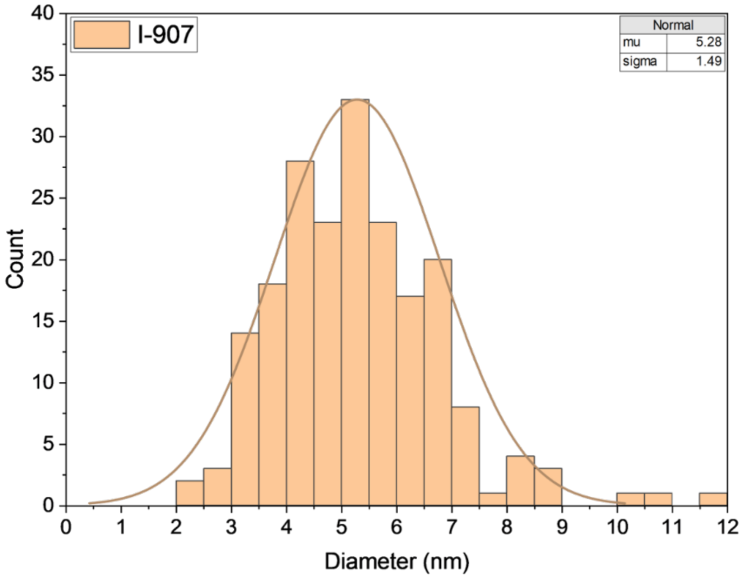

Figure S9**:** Top: Representative TEM image of AgNPs generated using the optimized flow reaction conditions using I-907 as a photoinitiator. Bottom: Corresponding histogram for I-907 generated AgNPs, n = 200 particles. Inset lists average size, mu, and standard deviation, sigma, in nm.

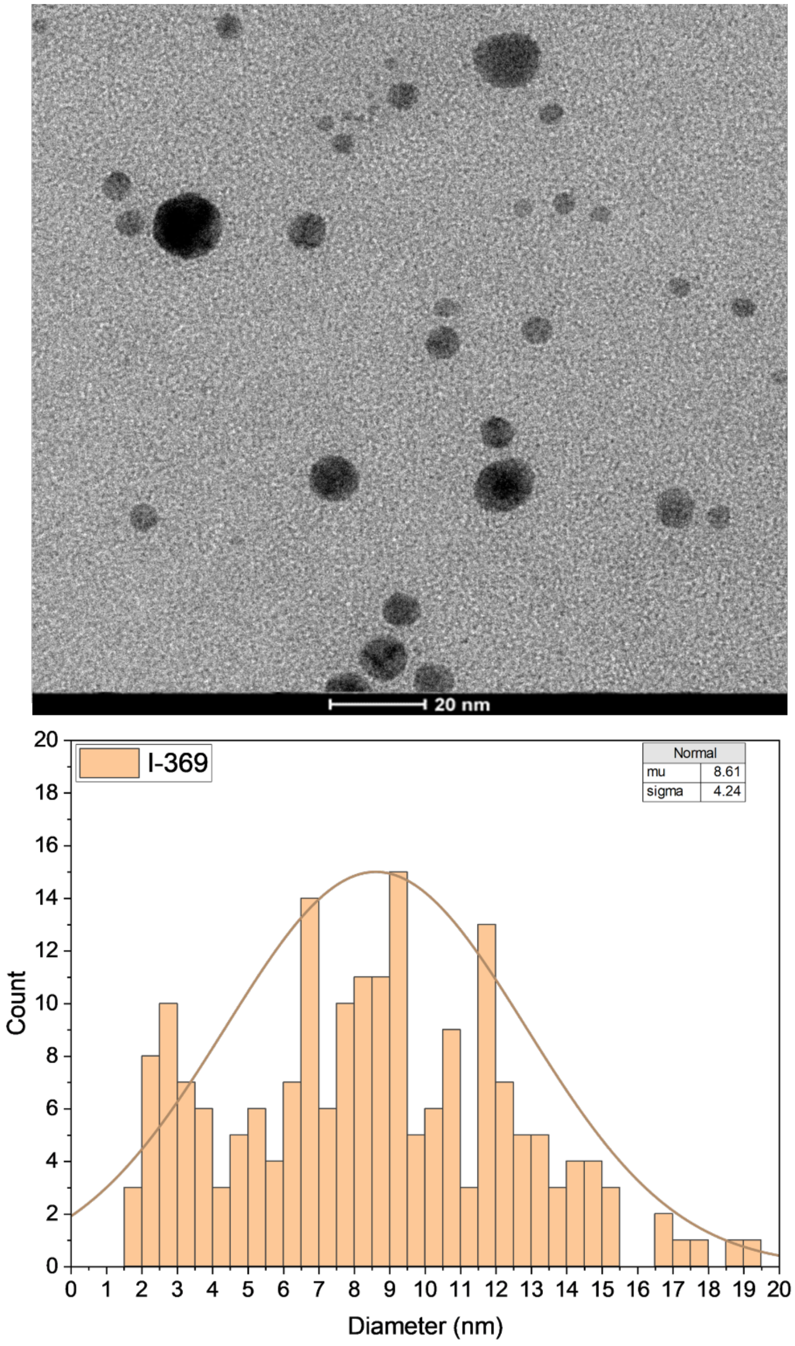

Figure S10: Top: Representative TEM image of AgNPs generated using the optimized flow reaction conditions using I-369 as a photoinitiator. Bottom: Corresponding histogram for I-369 generated AgNPs, n = 200 particles. Inset lists average size, mu, and standard deviation, sigma, in nm.

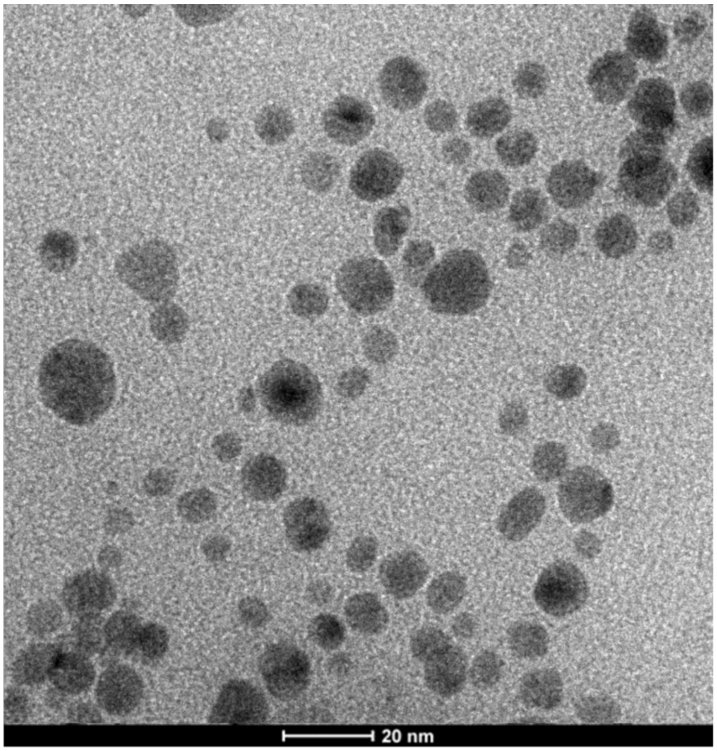

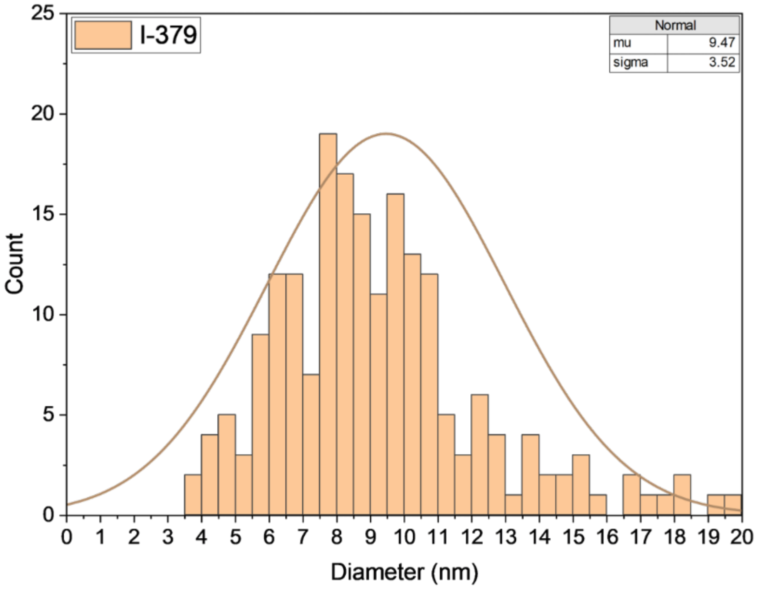

Figure S11: Top: Representative TEM image of AgNPs generated using the optimized flow reaction conditions using I-379 as a photoinitiator. Bottom: Corresponding histogram for I-379 generated AgNPs, n = 200 particles. Inset lists average size, mu, and standard deviation, sigma, in nm.

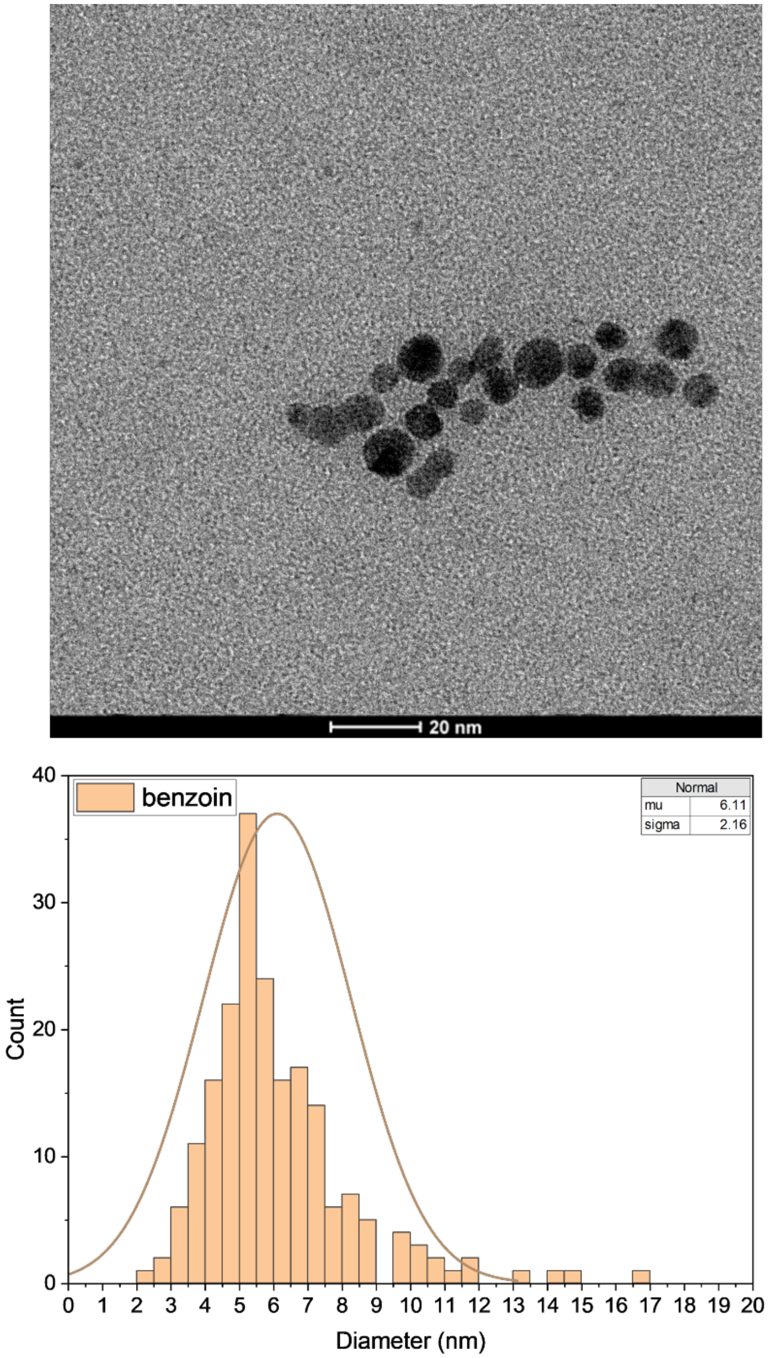

Figure S12**:** Top: Representative TEM image of AgNPs generated using the optimized flow reaction conditions using benzoin as a photoinitiator. Bottom: Corresponding histogram for benzoin generated AgNPs, n = 200 particles. Inset lists average size, mu, and standard deviation, sigma, in nm.

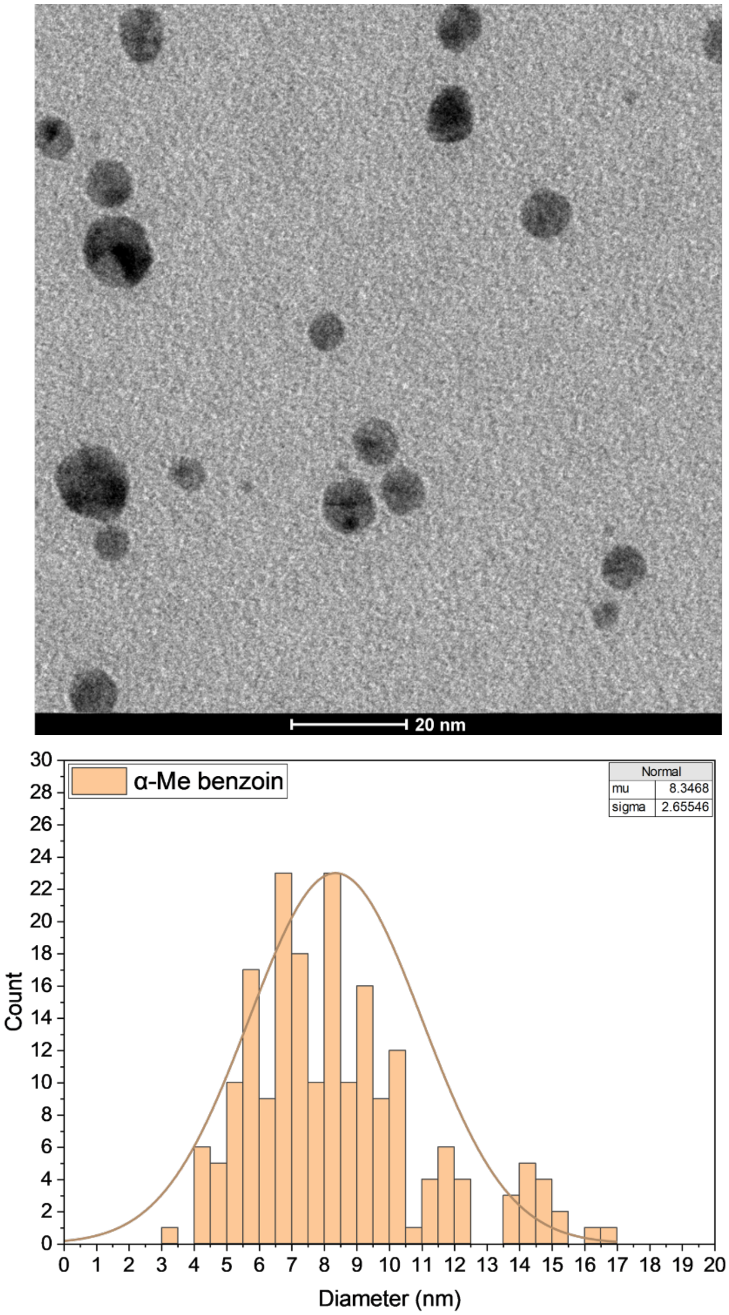

Figure S13**:** Top: Representative TEM image of AgNPs generated using the optimized flow reaction conditions using α-methylbenzoin as a photoinitiator. Bottom: Corresponding histogram for α-methylbenzoin generated AgNPs, n = 200 particles. Inset lists average size, mu, and standard deviation, sigma, in nm.

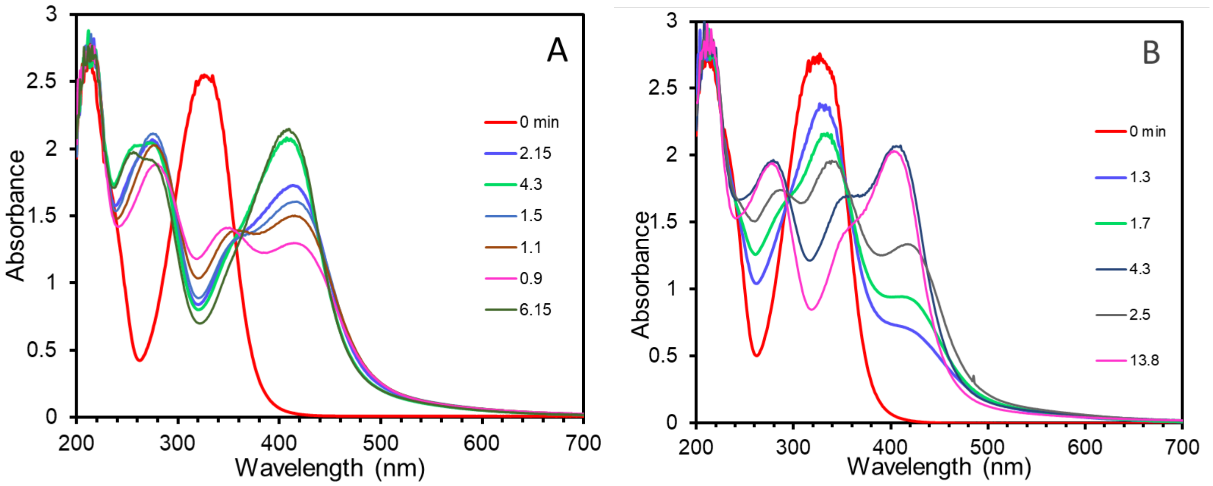

Figure S14**:** I-369 as an initiator for spectra corresponding to different residence times in minutes. Silver nitrate (0.2 mM), benzoin (0.2 mM), and trisodium citrate (1 mM) in 1:1 CH3OH: water deaerated with argon and irradiated in a flow system with 6.3 m Teflon tubing with (A) UV-B lamp and (B) UV-A lamp.

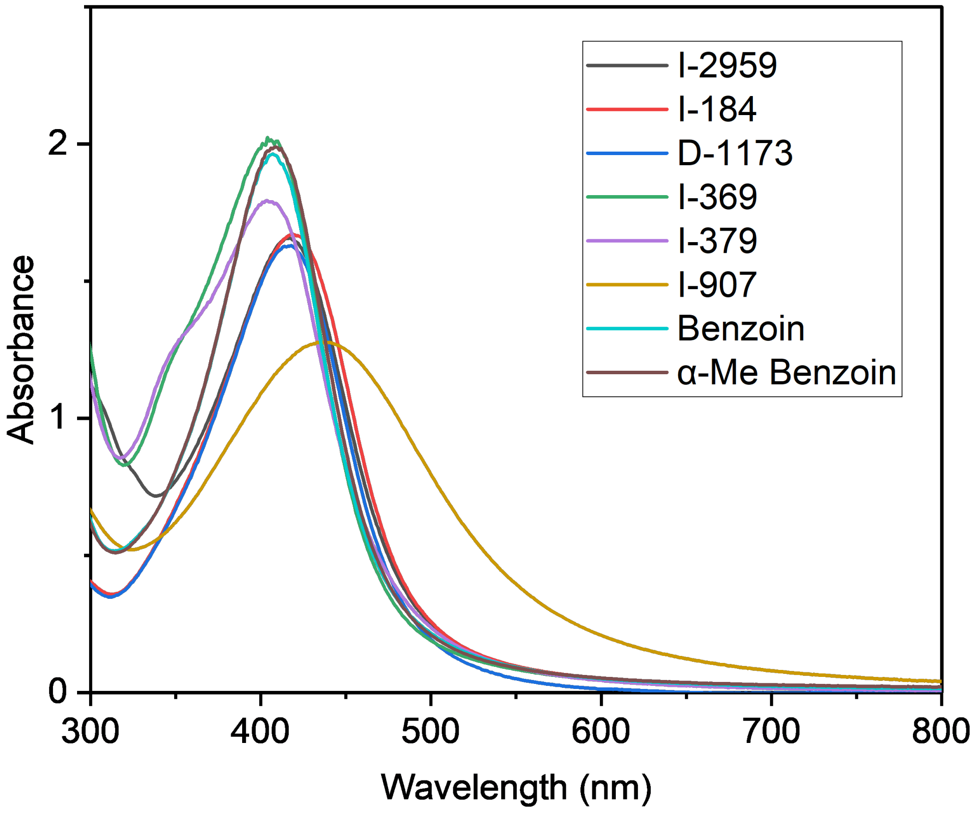

Figure S15**:** UV-Vis spectra of AgNPs made in-flow using 50% MeOH, 1 mM citrate, 0.2 mM AgNO3, 0.2 mM of photoinitiator under UVB irradiation. Flow rates for each are shown in Table 1. Corresponding TEM and histograms are shown in Figure S6-S13.
